# Oncogenic Roles of Polycomb Repressive Complex 2 in Bladder Cancer and Upper Tract Urothelial Carcinoma

**DOI:** 10.3390/biomedicines10112925

**Published:** 2022-11-14

**Authors:** Eric Yi-Hsiu Huang, Yu-Kuang Chen, Chen-Pu Ou, Yi-Ting Chen, Sung-Fang Chen, William J. Huang, Kung-Hao Liang

**Affiliations:** 1 Department of Urology, Taipei Veterans General Hospital, Taipei 112, Taiwan; 2 Department of Urology, College of Medicine and Shu-Tien Urological Research Center, National Yang Ming Chiao Tung University, Taipei 112, Taiwan; 3 Department of Surgery, Pingtung Veterans General Hospital, Pingtung 900, Taiwan; 4 Department of Pathology and Laboratory, Kaohsiung Veterans General Hospital, Kaohsiung 813, Taiwan; 5 Department of Biomedical Sciences, Chang Gung University, Taoyuan 333, Taiwan; 6 Kidney Research Center, Department of Nephrology, Linkou Medical Center, Chang Gung Memorial Hospital, Taoyuan 333, Taiwan; 7 Department of Chemistry, National Taiwan Normal University, Taipei 106, Taiwan; 8 Department of Medical Research, Taipei Veterans General Hospital, Taipei 112, Taiwan; 9 Institute of Biomedical Informatics, National Yang Ming Chiao Tung University, Taipei 112, Taiwan; 10 Institute of Food Safety and Health Risk Assessment, National Yang Ming Chiao Tung University, Taipei 112, Taiwan

**Keywords:** epigenomics, precision medicine, transitional cell carcinoma, multi-omics investigations

## Abstract

Cancers of the urinary tract are one of the most common malignancies worldwide, causing high morbidity and mortality, and representing a social burden. Upper tract urothelial carcinoma (UTUC) accounts for 5–10% of urinary tract cancers, and its oncogenic mechanisms remain elusive. We postulated that cancers of the lower and the upper urinary tract may share some important oncogenic mechanisms. Therefore, the oncogenic mechanisms discovered in the lower urinary tract may guide the investigation of molecular mechanisms in the upper urinary tract. Based on this strategy, we revisited a high-quality transcriptome dataset of 510 patients with non-muscle invasive bladder cancer (NMIBC), and performed an innovative gene set enrichment analysis of the transcriptome. We discovered that the epigenetic regulation of polycomb repressive complex 2 (PRC2) is responsible for the recurrence and progression of lower-track urinary cancers. Additionally, a PRC2-related gene signature model was discovered to be effective in classifying bladder cancer patients with distinct susceptibility of subsequent recurrence and progression (log-rank *p* < 0.001 and = 0.001, respectively). We continued to discover that the same model can differentiate stage T3 UTUC patients from stage Ta/T1 patients (*p* = 0.026). Immunohistochemical staining revealed the presence of PRC2 components (EZH2, EED, and SUZ12) and methylated PRC2 substrates (H3K27me3) in the archived UTUC tissues. The H3K27me3 exhibited higher intensity and area intensity product in stage T3 UTUC tissues than in stage Ta/T1 tissues (*p* = 0.006 and 0.015, respectively), implicating stronger PRC2 activity in advanced UTUC. The relationship between H3K27 methylation and gene expression is examined using correlations. The H3K27me3 abundance is positively correlated with the expression levels of CDC26, RP11-2B6, MAPK1IP1L, SFR1, RP11-196B3, CDK5RAP2, ANXA5, STX11, PSMD5, and FGFRL1. It is also negatively correlated with CNPY2, KB-1208A12, RP11-175B9, ZNF692, RANP8, RP11-245C17, TMEM266, FBXW9, SUGT1P2, and PRH1. In conclusion, PRC2 and its epigenetic effects are major oncogenic mechanisms underlying both bladder cancer and UTUC. The epigenetically regulated genes of PRC2 in urothelial carcinoma were also elucidated using correlation statistics.

## 1. Introduction

Urinary tract cancer is a malignant solid tumor, the majority of which occurs at the transitional epithelium of the upper and lower urinary tract and is referred to as urothelial carcinoma [1,2]. With an incidence of 26 per 100,000 person-years, it is one of the most common cancers worldwide [3]. Upper tract urothelial carcinoma (UTUC) accounts for 5–10% of all urinary tract cancers in Western countries [4,5,6]. Although UTUC accounts for only a small proportion of urinary cancers, its incidence is steadily increasing in countries such as the Netherlands [7]. The age-standardized incidence of UTUC in Taiwan is over 4 per 100,000 people [8]. In the lower tract, urothelial carcinoma of the bladder accounts for the majority (>90%) of all bladder cancers [8]. The incidence of urothelial carcinoma of the bladder appears to be stable in countries such as Norway [9], while it is increasing in United States, especially in men [10]. A comparative investigation showed that patients with urothelial carcinoma in the upper tract tend to have more advanced stages and poorer histopathologically confirmed cell differentiation than those in the lower tract [11]. In Asia, the incidence of UTUC is high [12,13]. Patients with end-stage renal disease are at higher risk of UTUC and lower tract urothelial carcinoma [14]. The proportion of female UTUC patients is higher in Asia than in Western countries [15]. The epidemiological distributions of the different types of urothelial carcinomas provides clues to their underlying molecular mechanisms.

Cancers in the upper and lower urinary tract share common [16] and distinct [6,17] molecular features. Activations of oncogenes, such as the tyrosine kinases hRAS and FGFR3, and defects of tumor suppressors, such as Tp53 and RB, are implicated for urothelial carcinomas of the bladder [18]. Tp53, MDM2, RAS, and FGFR3 can be used to derive UTUC subtypes with a different prognosis [19]. Epigenomic effects, such as histone modifications, are thought to be responsible for the occurrence and progression of many cancers [20], including urothelial carcinomas of the bladder [21,22] and UTUC [16]. Regarding the etiologies, exposure to tobacco smoke is associated with the occurrence of urothelial carcinoma [23]. This association is due to the tobacco smoke-elicited epigenomic effect [24]. In Taiwan, consumptions of drinking waters contaminated by arsenic [13] and herbal medicine made by Aristolochiaceae (birthworts; common ingredients of herbal medicines) [25] are implicated to cause the formation of DNA adducts, which in turn triggers carcinogenesis [26,27].

To date, the driving mechanism of UTUC remains largely elusive, probably due to the relatively low incidence and low number of study participants. Clarification of the driving mechanisms can help to achieve novel and personally optimized treatments of UTUC. Considering the intricate relationship between upper tract and lower urinary tract cancers, we postulated that prominent molecular signatures of urinary cancers in the lower tract may guide the research directions to decipher the oncogenic mechanisms of UTUC. Hence, we were intrigued to perform novel analysis of a high-quality public-domain dataset of 510 non-muscle invasive bladder cancer (NMIBC) patients [28]. We conducted an independent analysis focusing particularly on the shared mechanism underlying recurrence and progression, as well as the related predictive model, which can stratify patients with respect to their distinct risks of subsequent recurrence and progression. The prominent molecular signature elucidated by this analysis was used as clues for deciphering the driving mechanism of UTUC.

## 2. Materials and Methods

### 2.1. Analysis of Public-Domain Lower Trcat Urinary Cancer Dataset

We performed innovative analysis of a high-quality public-domain dataset of 510 NMIBC patients, comprising both the RNAseq transcriptomic profiles of surgical tumor tissues and the clinical time to recurrence and time to progression to muscle invasive bladder cancer (MIBC). This dataset is downloaded from the online Supplementary Source Data of Lindskrog et al. “https://www.nature.com/articles/s41467-021-22465-w#Sec38 (accessed on 26 October 2021)” [28].

### 2.2. Patients and Samples of Upper Tract Urinary Cancers

A collection of 36 archived surgical tissues of UTUC tumors were retrieved from the biobank of Taipei Veterans General Hospital, Taiwan. These tissues were frozen using liquid nitrogen shortly after surgical resections, and then transferred to −80 °C freezers for long-term storage in the biobank [29]. Part of the tissues were formalin-fixed and paraffin-embedded (FFPE), then sliced into 5μm slices for subsequent immunohistochemistry staining.

### 2.3. Immunohistochemistry Staining of Major Subunits of PRC2 and the Methylated Substrate H3K27me3

The immunohistochemistry staining assay was employed to examine the presence of three major components of the PRC2 complex, EZH2, EED, and SUZ12, as well as the methylated substrate H3K27me3, for checking the involvement of PRC2 in UTUC. The FFPE samples were deparaffinized, dehydrated, and incubated with the antibodies of EZH2 (concentration 1:50, Cell Signaling Technologies, Beverly, MA, USA, #5246), EED (1:100, Cell Signaling #85322), SUZ12 (1:100, Cell Signaling #3737) and H3K27me3 (1:200, Cell Signaling C36B11) for the staining. The staining was performed on the BOND-MAX autostainer (Leica, Wetzlar, Germany). The percentage of positively stained cells was determined by counting 100 cells in two fields.

### 2.4. Transcriptomic Profiling by RNA Sequencing

RNA was extracted from the 36 UTUC tumor samples using TRIzol LS reagent (Thermo Fisher Scientific, Wilmington, DE, USA). Concentrations were evaluated by Qubit (Thermo Fisher Scientific, Wilmington, DE, USA) and RNA size evaluated by Agilent Tapestation. The RNAseq technology was used for deep transcriptome profiling of the samples, including mRNA and long non-coding RNA (lncRNA). RNA was converted to double-strand cDNA as sequencing libraries. Primer dimers were eliminated using 1× Agencourt RNAClean XP beads after library preparation. Standard sequencing and analysis pipeline of the Illumina platform were employed. Data were analyzed using Burrows–Wheeler Aligner (BWA), SAMtools and the DESeq software package.

### 2.5. Statistical Analysis

Baseline demographic information was analyzed using Fisher’s exact test for categorical variables and independent *t*-test for continuous variables. Time-to-recurrence and progression were compared using log-rank test and visualized using Kaplan–Meier survival plots. Gene set enrichment analysis was performed for analyzing the comparative transcriptome, using a Java client software (version 4.2.3). The GSEA software is linked to the online Human Molecular Signatures Database (MSigDB), containing a comprehensive collection of reference gene sets. We used the oncogenic signature gene set (C6).

Gene signature risk models were derived using the generalized iterative modeling approach, a type of machine learning approach which performs empirical maximization with respect to either the log likelihood in the proportional hazards model [30] or the area under the receiver operating characteristic curve in classification analysis [31]. In this study, we selected to maximize the log likelihood in the time-to-MIBC survival analysis. The code can be found in the public domain GitHub site “https://github.com/khliang/GIM (accessed on 14 January 2022)”.

All statistical analyses were performed using SPSS package version 25.0 (IBM SPSS Inc., New York, NY, USA) and R (version 3.5.0). Significant differences were declared if the significance level *p* < 0.05.

## 3. Results

### 3.1. Polycomb Repressive Complex 2 Identified as a Major Driving Mechanism of Bladder Cancer Recurrence and Progression

We revisited a high-quality public-domain dataset of 510 NMIBC patients (Lindskrog et al., [28]). This dataset contains transcriptomic profiles of surgical tumor tissues, accompanied by the clinical outcome of the patients including the time to recurrence and time to progression, which is defined as the occurrence of MIBC. It is a valuable resource for deciphering mechanism of recurrence and progression. The five-year recurrence rate of this cohort is ~60%.

**Figure 1:**
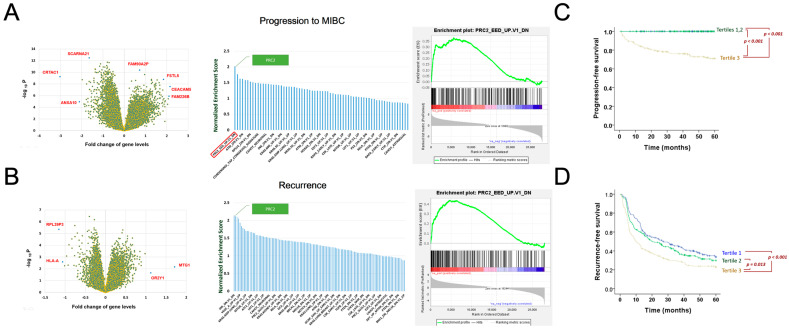
Discovery of PRC2 and downstream machinery, which drives the progression and recurrence of NMIBC after curative surgery. (**A**) The GSEA analysis of leading oncogenic mechanisms pertaining to the progression to MIBC, using transcriptome in patients with and without progression. The differential transcriptome is shown as a scatter plot of fold change and negative logarithm of significance (i.e., a volcano plot) in the left. Among a collection of 120 oncogenic mechanisms evaluated, the PRC2-related oncogenic mechanism is identified as the top driving mechanism with the highest normalized enrichment score (NES = 2.01). The standard GSEA enrichment plot of the PRC2-related oncogenic mechanism is shown in the right. (**B**) The GSEA analysis of leading oncogenic mechanisms pertaining to NMIBC recurrence, based on the differential transcriptome in patients with and without recurrence, which is shown as a volcano plot in the left. JNK and PRC2 are the top two major mechanisms involved, with a normalized enrichment score >2. One in four consecutive gene sets were shown in the x-axis label due to space limitation. (**C**) The Kaplan–Meier plot of time to progression (to MIBC) in patient strata by the risk model shown in Equation (1). Blue: tertile 1. Green: tertile 2. Brown: tertile 3 (overall log-rank *p* < 0.001). (**D**) The Kaplan–Meier plot of time to recurrence in patient strata by the risk model (overall log-rank *p* = 0.001).

We first compared the transcriptome of patients with and without the progression to MIBC (n = 45 and 465, respectively). Differential transcriptome is visualized by a volcano plot of individual genes based on the fold change and statistical significance of the comparison (Figure 1A left, Supplementary Table S1). Gene set enrichment analysis (GSEA) was then employed for assessing the importance of 189 major oncogenic mechanisms (collectively known as the C6 gene set collection of oncogenic signature) with respect to the progression to MIBC, using comparative transcriptome ranked by the t-statistics [32]. The polycomb repressive complex 2 (PRC2) and its downstream machinery is identified as the most important mechanism based on its highest normalized enrichment score (NES = 2.01, Figure 1A middle, Supplementary Table S2). The PRC2 gene set comprises a total of 193 PRC2 and its downstream genes including EED, an important component of the PRC2 protein complex. The PRC2 related genes all locate toward the leading edge of comparative transcriptome (Figure 1A right).

We also investigated the comparative transcriptome of recurrence, using the patient cohort excluding the 45 patients who developed MIBC during the follow up. This allows us to focus only on the transcriptome difference in patients with and without recurrence (n= 295 and 170, respectively, Figure 1B left, Supplementary Table S3). The GSEA analysis shows that JNK machinery and PRC2 machinery are the two most important oncogenic mechanisms involved (NES = 2.14 and 2.12, respectively, Figure 1B middle, Supplementary Table S4). The PRC2-related genes all locate toward the leading edge of comparative transcriptome (Figure 1B right). Since PRC2 manifested a shared mechanism underlying recurrence and progression, we postulated that the molecular machinery epigenetically regulated by PRC2 is responsible for the poor outcome of bladder cancer.

We then constructed a gene signature risk model to differentiate patients with distinct subsequent progression to MIBC based on the PRC2-related transcriptome. The risk score is defined as:

Score = GJC1 * (2.8819) + MKI67 * (1.3703) + ENSG00000237813 * (1.8403) + PCDHB3 * MTND1* (0.1520) + SPTB * NEAT1 * (0.1886) + LINC01285 * SPTB * CDC37L1 *(0.2124).(1)

The patients were stratified according to the tertiles of the gene signature score. Each strata correspond to 170 patients. The time to MIBC of the patient strata manifested different curves in the Kaplan–Meier plot (overall log-rank *p* < 0.001, Figure 1C). No patients in tertile 1 and 2 developed MIBC during the follow up. All the 45 MIBC patients appear in tertile 3, resulting in a significant difference of time to MIBC curves between tertile 3 and tertiles 1/2 (both *p* < 0.001). We then evaluated patient strata with respect to the time to recurrence. Significant differences are shown in the Kaplan–Meier plot in Figure 1D (overall log-rank *p* = 0.001)**.** The significant disparity of time to events in the score-stratified patient groups showed that the PRC-2-related gene signature score in Equation (1) can indicate subsequent recurrence and progression.

### 3.2. Evaluating PRC2 Oncogenic Signature in Asian Upper-Tract Urothelial Carcinoma Patients

**Figure 2:**
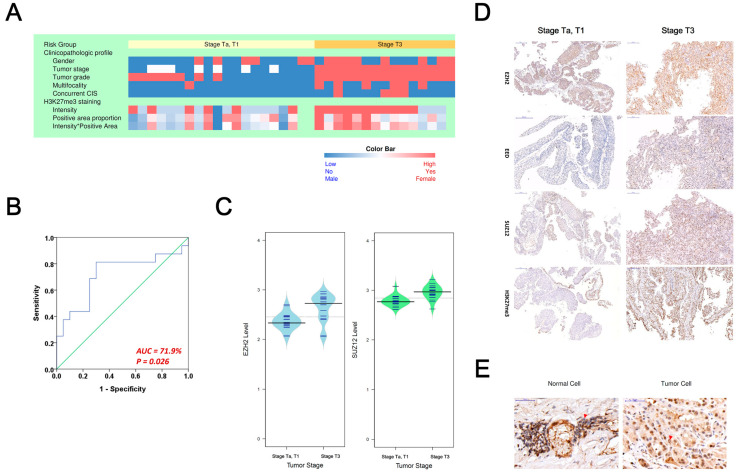
**An evaluation of PRC2 oncogenic signature in upper-tract urothelial carcinoma.** (**A**) The heatmap of clinicopathologic variables of UTUC patients and H3K27me3 staining intensities. (**B**) The performance of the risk model in Equation (1) for classifying advanced stage (n = 16) and low stage (n = 20) UTUC tissue samples, based on the risk score values calculated by the model with quantified RNA expression levels. (**C**) Distributions of EZH2 and SUZ12 levels in tissues with stage Ta/T1 or T3. (**D**) Representative immunohistochemical staining images of EZH2, EED, SUZ12, and H3K27me3 expressions in low stage and advanced stage UTUC. Magnification is ×100. (**E**) EZH2 protein expressions in normal urothelium tissue and UTUC at the 1:1 scale.

The role of the PRC2 oncogenic mechanism identified in the bladder cancer offers clues regarding the oncogenic mechanism in UTUC. We started by checking the performance of the gene signature in Equation (1) in the task of classifying UTUC tissues with or without advanced stages. We obtained a collection of 36 archived fresh-frozen UTUC tumor samples from the biobank of Taipei Veterans General Hospital (TVGH), Taiwan, including 16 tumor tissues at stage T3 (i.e., the advanced stage) and 20 tissues at stages of Ta/T1. This study was approved by the institutional review board of TVGH. The clinical and pathologic information of UTUC patients with or without advanced stages is shown as a heatmap in Figure 2A and Table 1**.** Basic demographic profiles, such as gender, age, and smoking status showed no significant difference between the two patient groups. On the other hand, tumor characteristics, such as multifocal and carcinoma in situ (CIS) are more prevalent in patients with advanced stages (*p* = 0.021 and 0.031, respectively). We performed RNAseq assay of the tumor tissues and calculate risk scores of the patients using the risk model in Equation (1). The result shows that the model can classify the patients with or without advanced stages successfully (area under the receiver operating characteristic curve = 71.9%, *p* = 0.026, Figure 2B).

EZH2, SUZ12, and EED are three major components of the PRC2 protein complex. This protein complex can regulate the methylation of histone proteins H3, at the lysine 27 (K27) position, which is denoted as H3K27. The protein complex mediates the methylation of histone protein H3K27 into H3K27me3, thereby regulating the downstream gene expressions. The EZH2 and SUZ12 expression levels, quantified by RNAseq, are shown as bean plots (Figure 2C). Both genes showed higher expression levels in tissues with stage T3 than Ta/T1 (*p* = 0.002 and 0.001, respectively). Additionally, we performed immunohistochemical staining of EZH2, EED, SUZ12, and H3K27me3 on archived UTUC tumor tissues to reveal the presence of PRC2 (Figure 2D). H3K27me3 and the three PRC2 proteins are positive in patients with or without advanced stages, but those patients with more advanced stages manifested stronger staining intensity (Figure 2D). The EZH2 protein in UTUC tissue and adjacent normal tissue are also shown (Figure 2E).

A total of 32 tissues were available for the H3K27me3 staining, and the intensity and percentage of H3K27me3 were used for statistical analysis. The staining intensity is significantly higher in advanced UTUC patients (intensity *p* = 0.006, intensity and area product *p* = 0.015, Table 1).

### 3.3. Deciphering Downsrtream Oncogenic Mechanism of PRC2 in UTUC Patients

**Figure 3:**
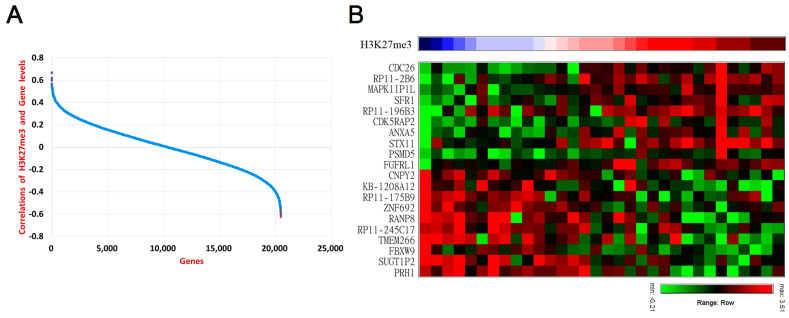
A screening of genes correlated with PRC2 abundance in UTUC. (**A**) Pearson’s correlations of H3K27me3 and transcriptomics. (**B**) Genes with the highest positive and negative correlations with H3K27me3 abundances.

The PRC2 complex is known to regulate downstream genes, primarily through the methylation of the histone protein H3K27, which in turn affects the expression of genes in close proximity. Methylation of histone in positions adjacent to genomic DNA promoters, insulators, enhancers, and transcribed regions may affect the regulation of gene expressions [33]. However, the genes regulated by PRC2 remain elusive in UTUC. To elucidate the PRC2 downstream oncogenic mechanism, we evaluated the correlation of H3K27me3 abundances in the UTUC tissues with the gene expressions (Figure 3A, Supplementary Table S5). The genes with the highest correlation with H3K27me3 in the positive and negative directions are supposedly regulated epigenetically. It was found that CDC26, RP11-2B6 [34], MAPK1IP1L, SFR1, RP11-196B3, CDK5RAP2, ANXA5, STX11, PSMD5, and FGFRL1 are positively correlated with H3K27me3 abundances (Figure 3B).

On the other hand, CNPY2, KB-1208A12, RP11-175B9, ZNF692, RANP8, RP11−245C17, TMEM266, FBXW9, SUGT1P2, and PRH1 are negatively correlated with H3K27me3 abundances (Figure 3B).

## 4. Discussion

Non-mutational epigenetic reprogramming was enlisted recently as one major hallmark of cancer [35]. Modifications of histones in the chromatins represent one major type of epigenetic regulation for downstream gene expressions [36]. This is achieved by acetylation, phosphorylation, and methylation of histone proteins, and these modifications affect chromatin structures and the binding affinity of chromatin-associated regulatory factors [37,38,39]. The altered gene expressions then affect cancer initiation and/or progression. Regulation by histone modification is less frequently investigated by scientists than genetic mutations and DNA modifications [40].

PRC2-mediated gene regulation is an endogenous machinery for biological developments, but is also involved in cancer [41,42,43,44,45,46,47,48,49,50,51,52]. Overexpression of EZH2 is reported in many malignancies, such as breast, ovarian, prostate, and bladder cancer [53,54,55,56]. EZH2 inactivation was also observed in hematological malignancies [57,58]. The pleotropic roles (including oncogenic and tumor-suppressive roles) of PRC2 manifested in different types of cancer may be due to the different sets of genes regulated [45]. Furthermore, EZH2 was found to be associated with aggressiveness of prostate and breast cancer [53,59].

PRC2 is estimated to regulate more than 10% of all human genes in different places at different times [43]. PRC2 inhibitors developed as anti-cancer therapies [46], which prevents the methylation of histones in certain places of the genome [33] and increases the expression of genes in these regions [33]. The regulation depends on the concentration of PRC2. It has context-dependent oncogenic and tumor-suppressive effects. Both loss-of-function and gain-of-function mutations were observed in PRC2 [45]. Gene expression and PRC2-mediated silencing are mutual exclusive events. When genes are expressing actively, the PRC2 cannot be recruited to this region [45]. As a result, the correlation of histone methylation and gene expression offers clues about their regulatory relationships.

The recruitment of PRC2 to its target DNA, also known as the polycomb response element [60], is also mediated by non-coding RNAs [43]. The long non-coding RNA nuclear paraspeckle assembly transcript 1 (NEAT1) is the scaffold for influencing the downstream expression of EZH2 [49]. NEAT1 enhances bladder cancer cell lines proliferation and migration, and suppresses apoptotic effects [61]. NEAT1 is also present in our PRC-2-related signature risk score (Equation (1)). Apart from NEAT1, the lncRNA HOTAIR is shown to facilitate PRC2 occupancy and H3K27me3 deposition to target genes within a 40 kB region of human chromosome 2 [60]. The noncoding RNA XIST was shown to mediate the PRC2-induced X chromosome inactivation [60]. The noncoding RNA Kcnq1ot1 was shown to mediate the PRC2 induced H3K27me3 deposition, as well as subsequent gene suppression in the 1-Mb region within the Kcnq1 domain of the mouse genome [60].

In this study, we demonstrated the PRC2 epigenetic effect underlying the recurrence of NMIBC cancer and the progression to MIBC. This PRC2-regulated oncogenic effect was discovered via an innovative analysis. In literature, correlation between elevated EZH2, SUZ12 and/or EED gene expression and poor prognosis of bladder carcinoma were reported [56,62,63,64]. EZH2 is regarded as a potential therapeutic target for bladder cancer [65].

Additionally, we showed the PRC2 mediated oncogenic effects in UTUC. A comparison of demographic and tumorigenic factors of our UTUC cohort showed that most demographic factors (gender and age) manifest no significant difference, while most tumorigenic factors and H3K27me3 abundance manifest a statistically significant difference between low-stage and high-stage patients. H3K27me3, the tri-methylated substrate of the PRC2 complex, is more prominent in UTUC tissues with more advanced stages. We demonstrated that the correlation between gene expressions and H3K27me3 abundances offer clues of their regulatory relationships. However, the sample size is small in our UTUC study (20 patients in the low-stage group and 16 patients in the high-stage group; Table 1). This is a major limitation of this study, and the statistics need to be interpreted with caution.

One future direction of this research is to find biomarkers in urine, which are more accessible than tissues for the convenience of clinical use. The PRC2-mediated mechanisms discovered in tissue may also manifest as perturbation signals in urine, i.e., the concept of liquid biopsy [66]. Urine biomarkers are more amenable than tissue biomarkers and cystoscopy, the current standard method for the monitoring of tumor recurrence events after treatments. Sediment cells, DNA, mRNA, and proteins in the urine are all used for the development of biomarkers [66]. Hence, it is warranted to investigate PRC2-related urine biomarkers in urinary tract cancer patient cohorts. We previously discovered potential urine protein biomarkers indicating the occurrence of bladder cancer, including afamin, adiponectin, complement C4 gamma chain, apolipoprotein A-II precursor, ceruloplasmin, and prothrombin using multiplex reaction monitoring mass spectrometry (MRM-MS) [67]. It is our future research to validate these biomarkers and PRC2-related biomarkers, using urine samples of the independent patient cohort.

## 5. Conclusions

The PRC2 and its epigenetic effects were discovered as major oncogenic mechanisms of bladder cancer and UTUC. PRC2 mediated the methylation of H3K27 into H3K27me3, the abundance of which indicates the stages of UTUC. The epigenetically regulated genes were also elucidated using correlation statistics.

## Figures and Tables

**Table 1:** Clinical, pathological and H3K27me3 staining value distributions.

	Tumor Stage Ta/T1	Tumor Stage T3	*p* Value
Number	20	16	
Gender			0.190
Male	14 (70%)	8 (50%)	
Female	6 (30%)	8 (50%)	
Age	62.25 ± 13.93	62.56 ± 8.18	0.934
Hydronephrosis	12 (60%)	13 (81%)	0.156
Smoking	8 (40%)	2 (13%)	0.071
Hypertension	9 (45%)	4 (25%)	0.187
Diabetes	7 (35%)	2 (7.0%)	0.122
Coronary artery disease	3 (15%)	0 (0.0%)	0.160
ECOG			0.577
0	8 (40%)	6 (37%)	
1	12 (60%)	10 (63%)	
Location			0.090
Renal pelvis	10 (53%)	11 (69%)	
Ureter	9 (47%)	3 (19%)	
Renal pelvis + ureter	0 (0.0%)	2 (13%)	
Morphology			0.444
Papillary	20 (100.0%)	15 (94%)	
Non-papillary	0 (0.0%)	1 (6%)	
Pathological T stage			**<0.001**
Ta	13 (65.0%)		
T1	7 (35.0%)		
T3		16 (100%)	
Tumor grade			**<0.001**
Low grade	12 (60%)	0 (0%)	
High grade	8 (40%)	16 (100%)	
Tumor size			**0.007**
<3 cm	11 (55.0%)	2 (13%)	
≧3 cm	8 (40.0%)	14 (88%)	
Multifocal	1 (5.0%)	6 (37.5%)	**0.021**
Concurrent CIS	0 (0.0%)	4 (25%)	**0.031**
Lymphovascular invasion	0 (0.0%)	2 (13%)	0.190
H3K27me3 staining			
Intensity	2.17 ± 0.79	2.79 ± 0.43	**0.006**
Positive area proportion	0.63 ± 0.27	0.78 ± 0.13	0.094
Intensity × Area	1.50 ± 0.83	2.20 ± 0.57	**0.015**

## Data Availability

The data have been provided as Supplementary Tables of this manuscript.

## Supplementary Materials

The following supporting information can be downloaded at: https://www.mdpi.com/article/10.3390/biomedicines10112925/s1, Table S1: The comparative transcriptome of bladder cancers with or without the progression to MIBC. Table S2: Results of Gene Set Enrichment Analysis using the comparative transcriptome of bladder cancers with or without the progression to MIBC. Table S3: The comparative transcriptome of bladder cancers with or without the recurrence. Table S4: Results of Gene Set Enrichment Analysis using the comparative transcriptome of bladder cancers with or without the recurrence. Table S5: The transcriptome and HeK27me3 abundance in UTUC tissues.
